# The mitochondrial fission factor FIS1 promotes stemness of human lung cancer stem cells via mitophagy

**DOI:** 10.1002/2211-5463.13207

**Published:** 2021-06-19

**Authors:** Doudou Liu, Zhiwei Sun, Ting Ye, Jingyuan Li, Bin Zeng, Qiting Zhao, Jianyu Wang, Hongmei Rosie Xing

**Affiliations:** ^1^ State Key Laboratory of Ultrasound Engineering in Medicine Co‐Founded by Chongqing and the Ministry of Science and Technology School of Biomedical Engineering Chongqing Medical University China; ^2^ Institute of Life Sciences Chongqing Medical University China

**Keywords:** cancer stem cell, *Fis1*, FIS1, mitochondrial fission, mitophagy, stemness

## Abstract

Mitophagy, a form of autophagy, plays a role in cancer development, progression and recurrence. Cancer stem cells (CSCs) also play a key role in these processes, although it not known whether mitophagy can regulate the stemness of CSCs. Here, we employed the A549‐SD human non‐small cell lung adenocarcinoma CSC model that we have developed and characterized to investigate the effect of mitophagy on the stemness of CSCs. We observed a positive relationship between mitophagic activity and the stemness of lung CSCs. At the mechanistic level, our results suggest that augmentation of mitophagy in lung CSCs can be induced by FIS1 through mitochondrial fission. In addition, we assessed the clinical relevance of FIS1 in lung adenocarcinoma using The Cancer Genome Atlas database. An elevation in FIS1, when observed together with other prognostic markers for lung cancer progression, was found to correlate with shorter overall survival.

## Graphical abstract

In the human non‐small cell lung adenocarcinoma cancer stem cell model A549‐SD, inhibiting the expression of FIS1 can inhibit mitochondrial division, thereby inhibiting mitophagy and consequently inhibiting the stemness of cancer stem cells.

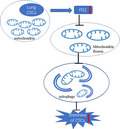

**Abbreviations**
CCCP: carbonyl cyanide *m*‐chlorophenyl hydrazone
CSC: cancer stem cell
Mdivi‐1: mitochondrial division inhibitor 1
ROS: reactive oxygen species
RT‐PCR: reverse transcriptase PCR
shRNA: small hairpin RNA
TCGA: The Cancer Genome Atlas

Cancer stem cells (CSC) were first discovered in the hematopoietic system [1] and subsequently identified in a broad spectrum of solid tumors. CSCs comprise a group of cells with self‐renewal and differentiation ability. They play a key role in tumorigenesis, metastasis and recurrence, as well as in the development of drug resistance [2, 3, 4, 5, 6, 7, 8, 9].

Autophagy is an evolutionarily conserved biological process of energy metabolism. By degrading intracellular organelles and proteins, autophagy provides cells with biochemical reaction substrates for the maintenance of homeostasis under nutrient deprivation or other stressful conditions [10]. Autophagy is mainly divided into macroautophagy, small autophagy and selective autophagy, among which macroautophagy has been the focus of the field [11]. The key processes of autophagy are the formation of a bilayer membrane structure in the cytoplasm by autophagy‐related proteins, bilayer membrane packaging of the substances that need to be degraded, the transportation of the contents of autophagic vesicles to the lysosome to form autophagosomes and, finally, the degradation of vesicle contents in the acidic environment of lysosomes [12]. Autophagy is an important metabolic form for normal cells. Researchers have reported that hematopoietic stem cells lost their ability to self‐renew and regenerate after inhibition of autophagy [13]. Autophagy is essential for the maintenance of stemness of both stromal stem cells [14] and embryonic stem cells [15].

The role of autophagy in cancer is context‐dependent. In the early stage of tumorigenesis, autophagy inhibits the development of tumors [16, 17], with autophagy‐related gene *beclin1* acting as a tumor suppressor [18]. In the metastasis stage of cancer, autophagy augments tumor progression [19]. Emerging evidence supports a role of autophagy in the regulation and maintenance of the stemness of CSCs. The level of basal autophagic activity is higher in CSCs than that in normal tumor cells, and changing the autophagy level will affect the stemness of CSCs [20]. Liu *et al*. [21] found that mitophagy maintains the hepatic CSC population by removing mitochondria‐associated p53, which otherwise would be activated by PINK1 (i.e. phosphatase and tensin homolog‐induced putative kinase 1) to suppress the expression of NANOG. However, a mechanistic understanding of the underlying autophagy regulation of the stemness of CSCs remains elusive.

Mitophagy is a form of selective autophagy that degrades damaged and aged mitochondria [22]. There is a strong correlation between mitochondrial division and the level of mitophagic activity. After mitochondrial division, senescent and damaged mitochondria are split. Thereafter, the damaged mitochondria are degraded by mitophagy and the excess reactive oxygen species (ROS) in the cell is metabolized at the same time [23]. Mitochondrial division is regulated by a number of mitochondrial fusion and fission genes, of which the most studied are the mitochondrial fission gene *Drp1* and the mitochondrial fusion gene *Opa1* [24]. Yamada *et al*. [25] showed that, in non‐alcoholic fatty liver, mitophagy of hepatocytes augments the progression of the disease. The mitophagic activity of hepatocytes depends on the size of mitochondria, which appears to be regulated by *Opa1* and *Drp1*. However, recent studies have shown that mitochondrial fission gene *Fis1*, encoding the FIS1 protein anchored on the outer membrane of the mitochondria, regulates mitochondrial division and the degradation of damaged mitochondria [26, 27].

Mitochondria act as an energy supply of cells, and mitophagy is also important for the survival of tumor cells and CSCs [28, 29]. A few recent studies have begun to show how mitophagy regulates the stemness of CSCs [30]. Pei *et al*. [31] showed that, in leukemia stem cells, FIS1 is highly expressed and FIS1‐mediated mitophagy is required for the maintenance of the stemness of leukemia stem cells. However, whether FIS1 directly regulates mitophagy is not clear. During our characterization of mitophagic activities in lung adenocarcinoma A549‐SD CSCs, we observed augmentation of stemness by mitophagy and the elevated expression of FIS1. The main finding derived from the present study is that FIS1 regulates mitophagy via mitochondrial division to promote the stemness of human lung CSCs.

## Results

### Mitophagy promotes the stemness of cancer stem cells

**Fig. 1:**
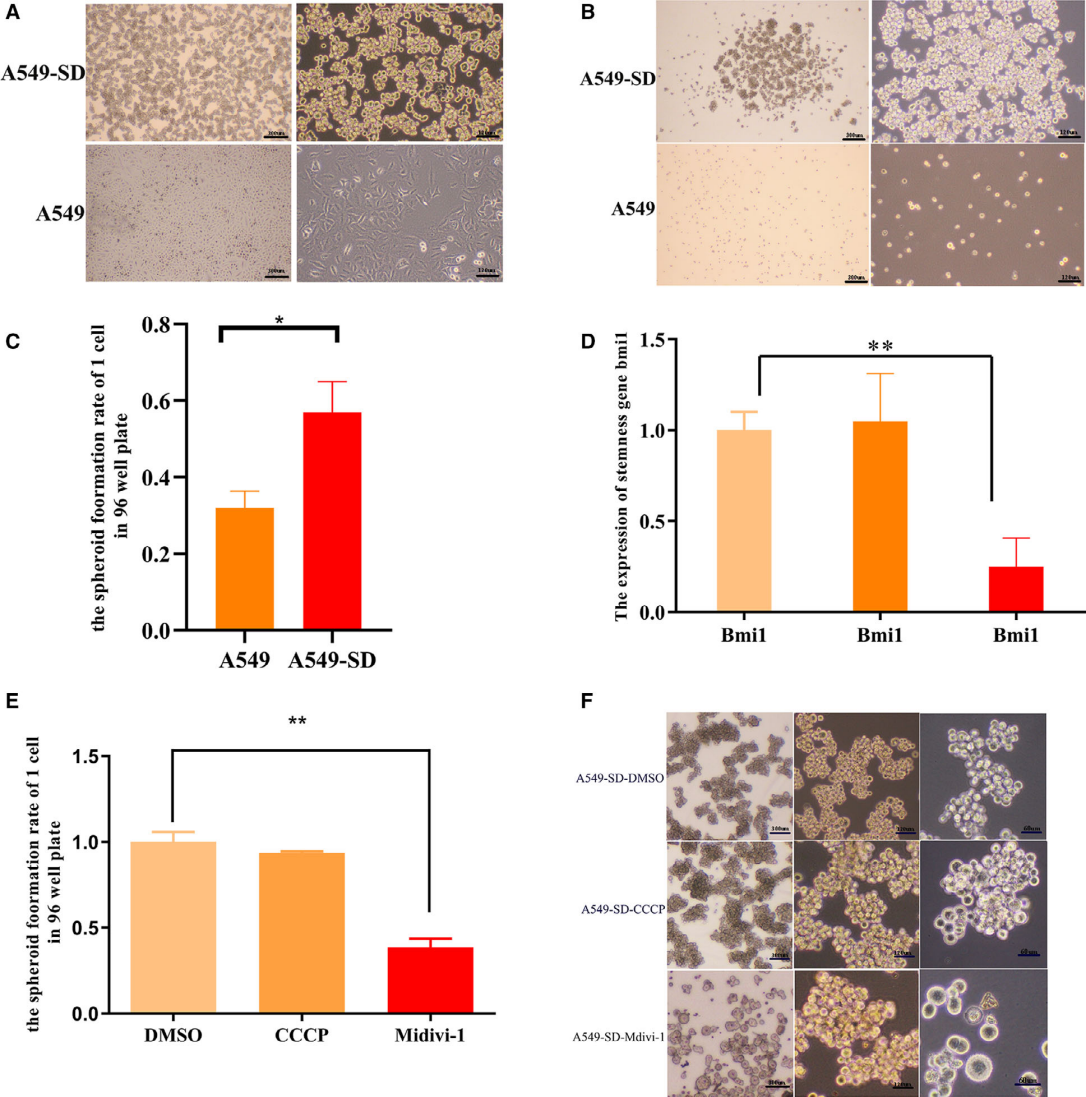
Mitophagy promotes the stemness of CSCs. (A) Morphological comparison of A549 cells and A549‐SD cells. The scale bars of the 4× and 10× images are 300 µm and 120 µm. (B) The spheroid formation ability of 1000 cells of A549 and A549‐SD cells in a six‐well plate. The scale bars of the 4× and 10× images are 300 µm and 120 µm. (C) The spheroid formation rate of a single cell in 96‐well plate of A549 and A549‐SD cells. (D) mRNA expression of stemness gene *bmi1* in DMSO, CCCP and Mdivi‐1 treated cells. (E) The spheroid formation rate of a single cell in a 96‐well plate of DMSO, CCCP and Mdivi‐1 treated cells. (F) The spheroid formation ability of 1000 cells of DMSO, CCCP and Mdivi‐1 treated cells in a six‐well plate. The scale bars of 4×, 10× and 20× images are 300, 120 and 60 µm, respectively. *P < 0.05, **P < 0.01; one‐way analysis of variance; the error bars indicate the SD; the number of biologically‐independent replicates is 3.

For the present study, we employed the A549‐SD human non‐small cell lung adenocarcinoma CSC model that we have developed and characterized In a previous study conducted in our laboratory, the stemness of A549‐SD cells was confirmed both *in vitro* and *in vivo* [32]. Before carrying out this research, we conducted some *in vitro* experiments to verify the stemness of A549‐SD lung CSCs. Although the parental A549 cells grow adherently, A549‐SD CSC cells grow in suspension (Fig. 1A). The results of the spheroid formation assay using 1000 cells in a six‐well plate and the spheroid formation assay of single cells in a 96‐well plate also showed that the spheroidization ability of A549‐SD cells was stronger than that of the parental A549 cells (Fig. 1B,C). To verify whether mitophagy affects the stemness of CSCs, we changed the level of mitophagic activity by treating human lung CSC A549‐SD with (a) carbonyl cyanide *m*‐chlorophenyl hydrazone (CCCP) that induces mitophagy and (b) mitochondrial division inhibitor 1 (Mdivi‐1) that inhibits mitophagy. These two drugs are classical drugs for mitophagy research [33, 34]. After 24 h of treatment, mRNA expression of the stemness gene was examined (Fig. 1D). The expression of *bmi1* was significantly decreased upon inhibition of mitophagy by Mdivi‐1. To further verify the effect of mitophagy on the stemness, we performed the single‐cell spheroid formation assay in a 96‐well plate and the consecutive spheroid formation assay using 1000 cells in a six‐well plate to measure spheroid formation efficiency. Inhibition of mitophagy upon treatment with Mdivi‐1 significantly decreased spheroid formation (Fig. 1E,F). The lack of robust effect of mitophagy induction by CCCP on the stemness of A549‐SD (Fig. 1) could be ascribed to the significantly enhanced stemness in A549‐SD cells as a result of CSC enrichment. Taken together, mitophagy activity is required for the maintenance of the stemness of A549‐SD lung CSCs.

### The mitochondria of non‐CSC tumor cells differ from those of the CSCs

**Fig. 2:**
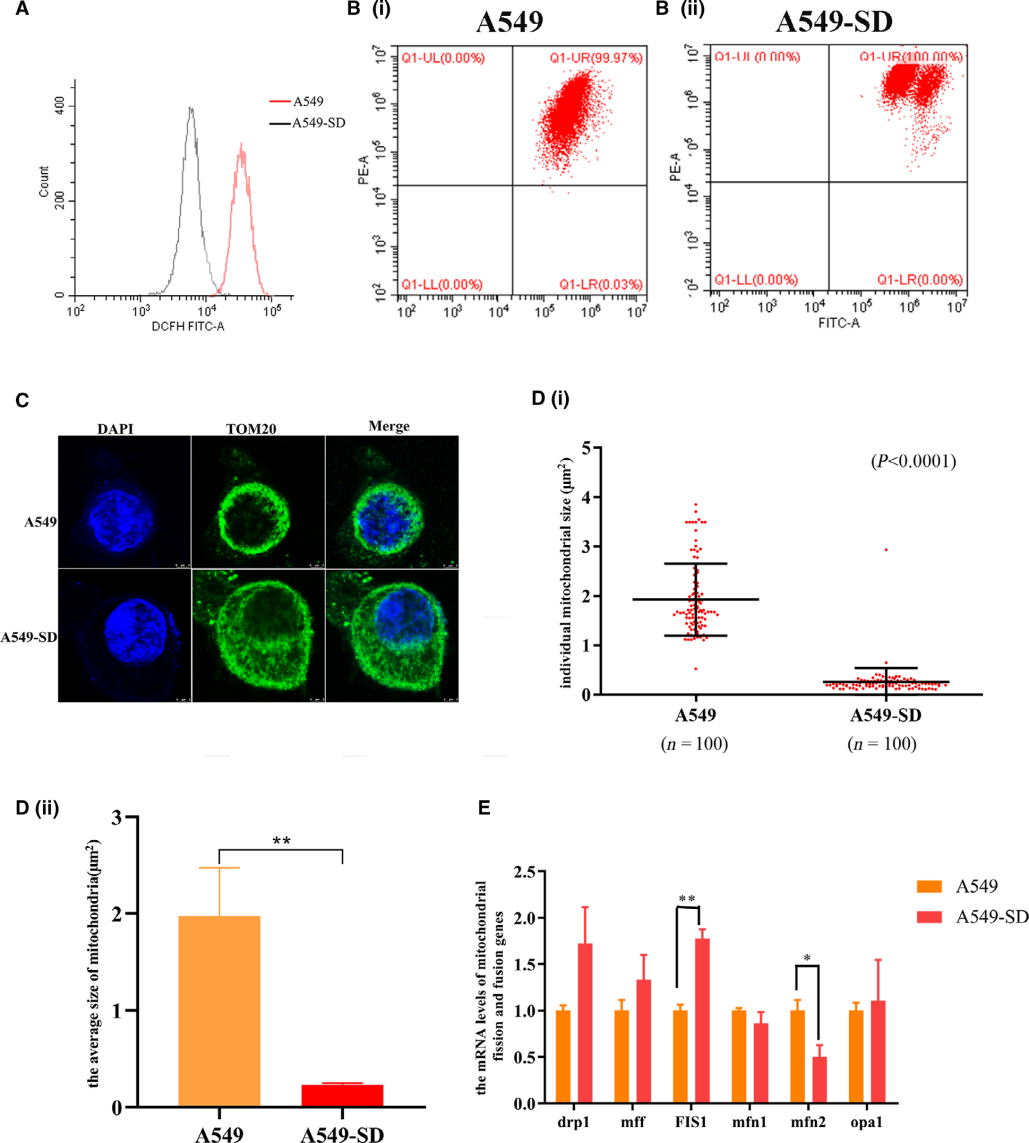
Differences between mitochondria in A459 parental and A549‐SD stem cells. (A) The ROS levels of A549 and A549‐SD cells. (Bi) The mitochondrial membrane potential of A549 cells. (Bii) The mitochondrial membrane potential of A549‐SD cells. (C) Distribution and size of mitochondrial in A549 and A549‐SD cells. Mitochondrial protein TOMM20 was used as a marker of mitochondrial. Scale bar = 3 µm. (Di) Sizes of 100 single mitochondria in A549 and A549‐SD cells. (Dii) The average size of 100 mitochondria in A549 and A549‐SD cells. (E) Expression of mitochondrial fission and fusion‐related genes in A549 and A549‐SD cells. *P < 0.05, **P < 0.01; one‐way analysis of variance; the error bars indicate the SD; the number of biologically‐independent replicates is 3.

A study reported that the levels of autophagy and the distribution of organelles are different in tumor cells and CSCs [35]. To explore the mechanisms underlying the regulation of the stemness of CSCs by mitophagy, we compared the differences in mitochondrial function between A549 and A549‐SD CSCs. We found that (a) A549‐SD CSCs have lower levels of ROS (Fig. 2A) and a higher membrane potential than A549 cells (Fig. 2Bi,ii). (b) Confocal immunofluorescence analysis revealed that the number of mitochondria in CSCs, visualized using TOM20 mito‐tracker (Proteintech, Chicago, IL, USA), was much higher than that of A549 cells (Fig. 2C). (c) The size of individual mitochondria was counted using imagej (NIH, Bethesda, MD, USA). We found that the mitochondria of non‐CSC tumor cells were larger than those of the CSCs (Fig. 2Di,ii). (d) The expression of the mitochondrial fission gene *Fis1* was most significantly increased in A549‐SD CSCs (Fig. 2E). This is consistent with the fact that mitochondrial content in A549‐SD CSCs is higher than that in A549 cells. We next examined the relationship between mitochondrial division and mitophagy and the stemness of CSCs, respectively.

### Mitochondrial division promotes mitophagy of cancer stem cells

**Fig. 3:**
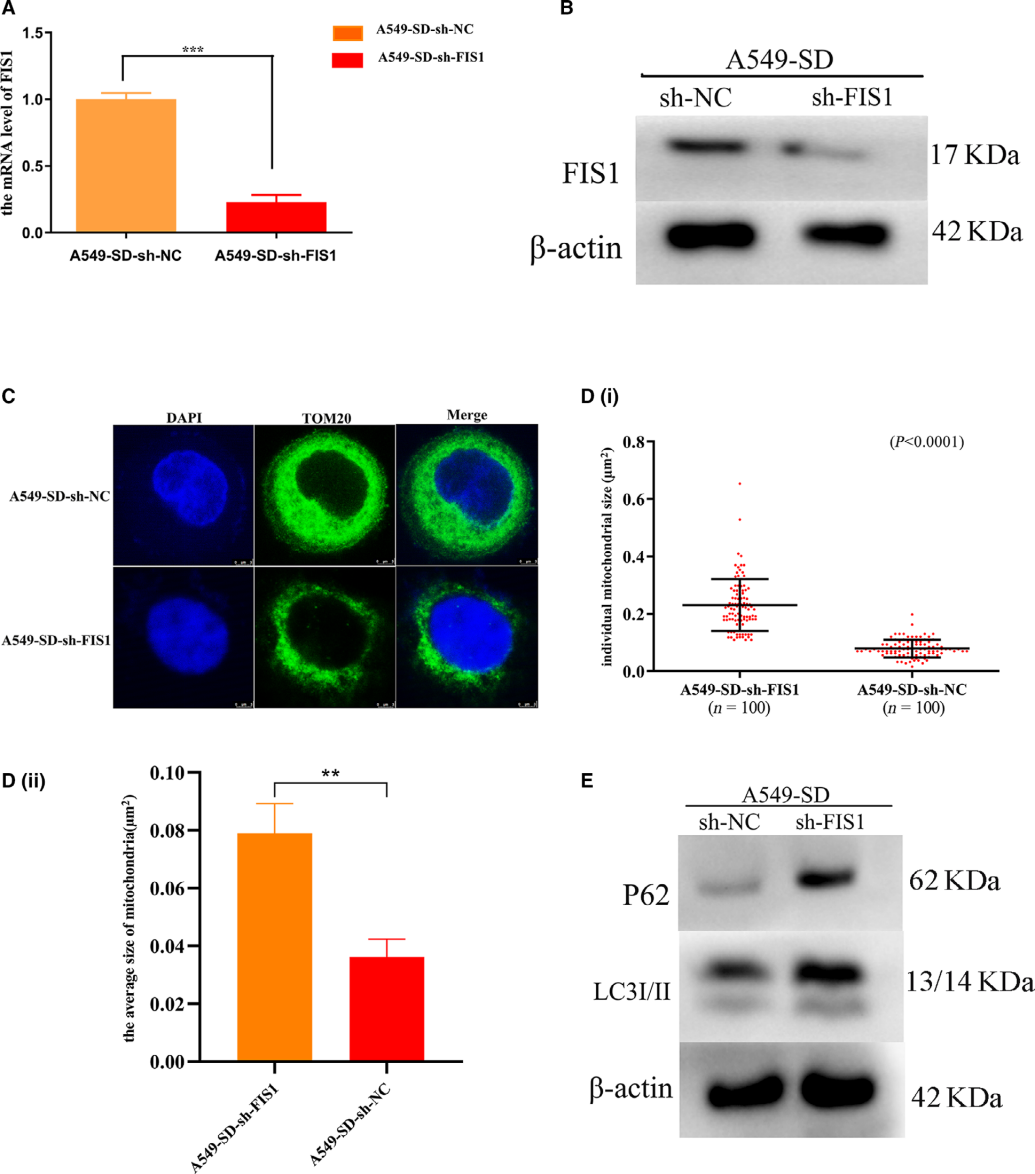
Mitochondrial division promotes mitophagy. (A) mRNA levels of FIS1 after sh‐mediated silencing of *Fis1*. (B) Expression of FIS1 protein after sh‐mediated silencing of *Fis1*. (C) Distribution and size of mitochondria after sh‐mediated silencing of *Fis1*. Scale bar = 3 µm. (Di) Sizes of 100 single mitochondria in A549‐SD‐sh‐NC and A549‐SD‐sh‐FIS1. (Dii) The average size of 100 mitochondria in A549‐SD‐sh‐NC and A549‐SD‐sh‐FIS1. (E) Expression of autophagy marker proteins P62 and LC3. **P < 0.01, ***P < 0.001; one‐way analysis of variance; the error bars indicate the SD; the number of biologically‐independent replicates is 3.

To explore whether mitochondrial division promotes mitophagy in A549‐SD CSCs, we used small hairpin RNA (shRNA)‐mediated knockdown of the mitochondrial division gene *Fis1* Fig. 3A,B) and examined the change in the mitochondria of A549‐SD CSCs. Upon knocking down *Fis1*, although the size of the mitochondria increased, the number decreased (Fig. 3C,Di,ii), indicating the successful blockade of the mitochondrial division. To determine the effect of changes in mitochondrial division rate on mitophagy, we examined the autophagy marker proteins P62 and LC3. After knocking down *Fis1*, accumulation of P62 was enhanced, whereas the conversion of LC3I to LC3II was attenuated (Fig. 3E). These observations indicate that FIS1 may be a key factor in activating autophagy.

### Mitochondrial fission promotes the stemness of CSCs

**Fig. 4:**
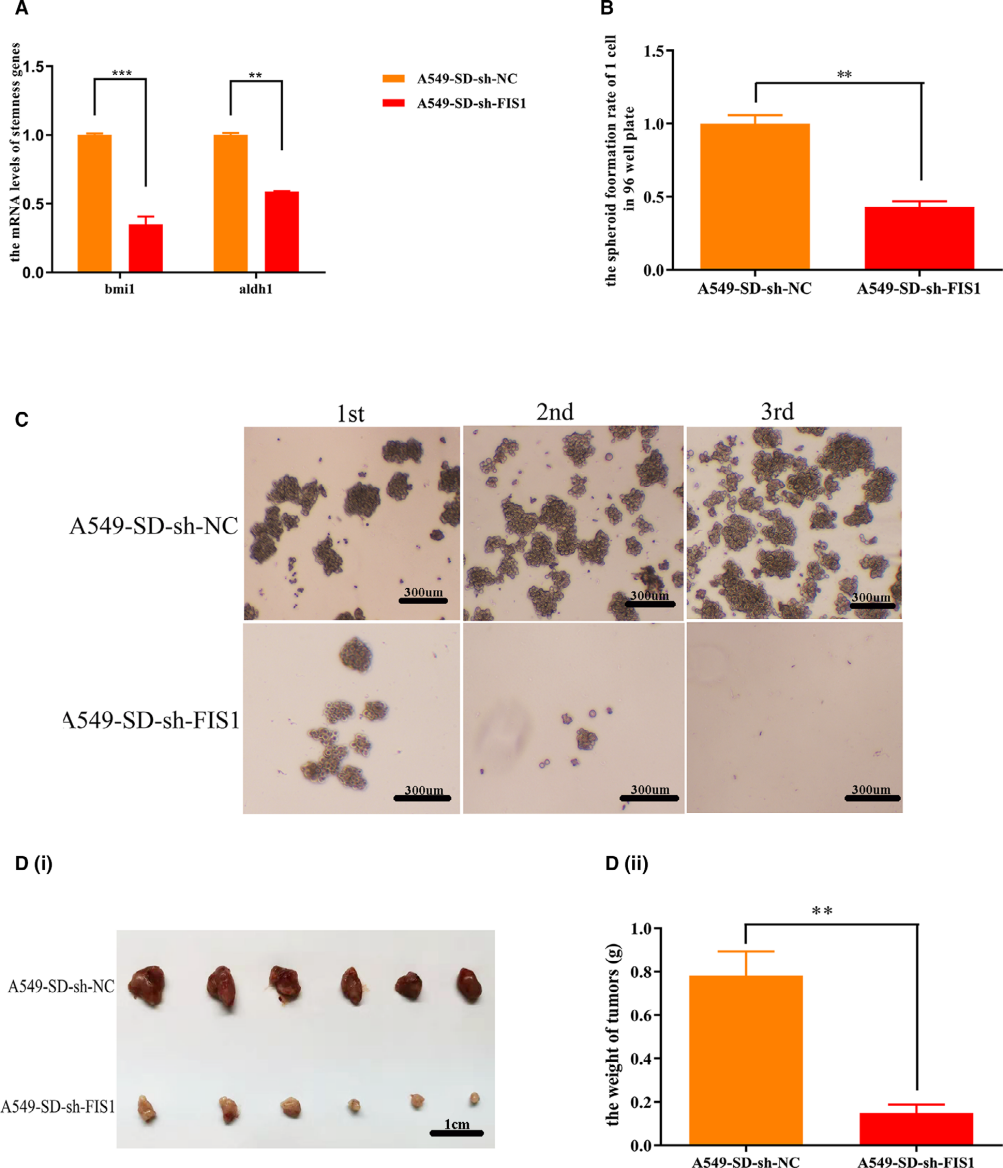
Mitochondrial division promotes the stemness of tumor stem cells. (A) Expression of stemness genes after sh‐mediated silencing of *Fis1*. (B) Spheroid formation rate of a single cell in a 96‐well plate after sh‐mediated silencing of *Fis1*. (C) Spheroid formation of 1000 cells in a six‐well plate after sh‐mediated silencing of *Fis1*. Scale bar = 300 μm. (Di) Tumors taken from nude mice 2 weeks after injection with A549‐SD‐sh‐NC and A549‐SD‐sh‐FIS1 cells. Scale bar is 1 cm. (Dii) Tumor weight of A549‐SD‐sh‐NC and A549‐SD‐sh‐FIS1 tumors. **P < 0.01, ***P < 0.001; one‐way analysis of variance; the error bars indicate the SD; the number of biologically‐independent replicates is 3.

To examine the effects of mitochondrial division on the stemness of A549‐SD CSCs, we performed a series of experiments *in vitro*. Knocking down of *Fis1* lead to a significant reduction of: (a) the expression of two key stemness genes, *bmi1* and *aldh1* (Fig. 4A) and (b) the spheroid formation efficiency (Fig. 4B,C) *in vitro*. *In vivo*, we performed a subcutaneous tumor transplantation assay in nude mice to determine the effect of *Fis1* knockdown on tumorigenesis efficiency. Accordingly, 100 000 tumor cells were transplanted subcutaneously on each side of the mouse and tumors were removed after 2 weeks of transplantation. Tumor formation was significantly reduced after *Fis1* interference (Fig. 4Di,ii).

Collectively, we concluded that mitochondrial division augments the stemness of CSCs by promoting mitophagy.

### The expression of FIS1 is lower in the early stage of lung cancer

**Fig. 5:**
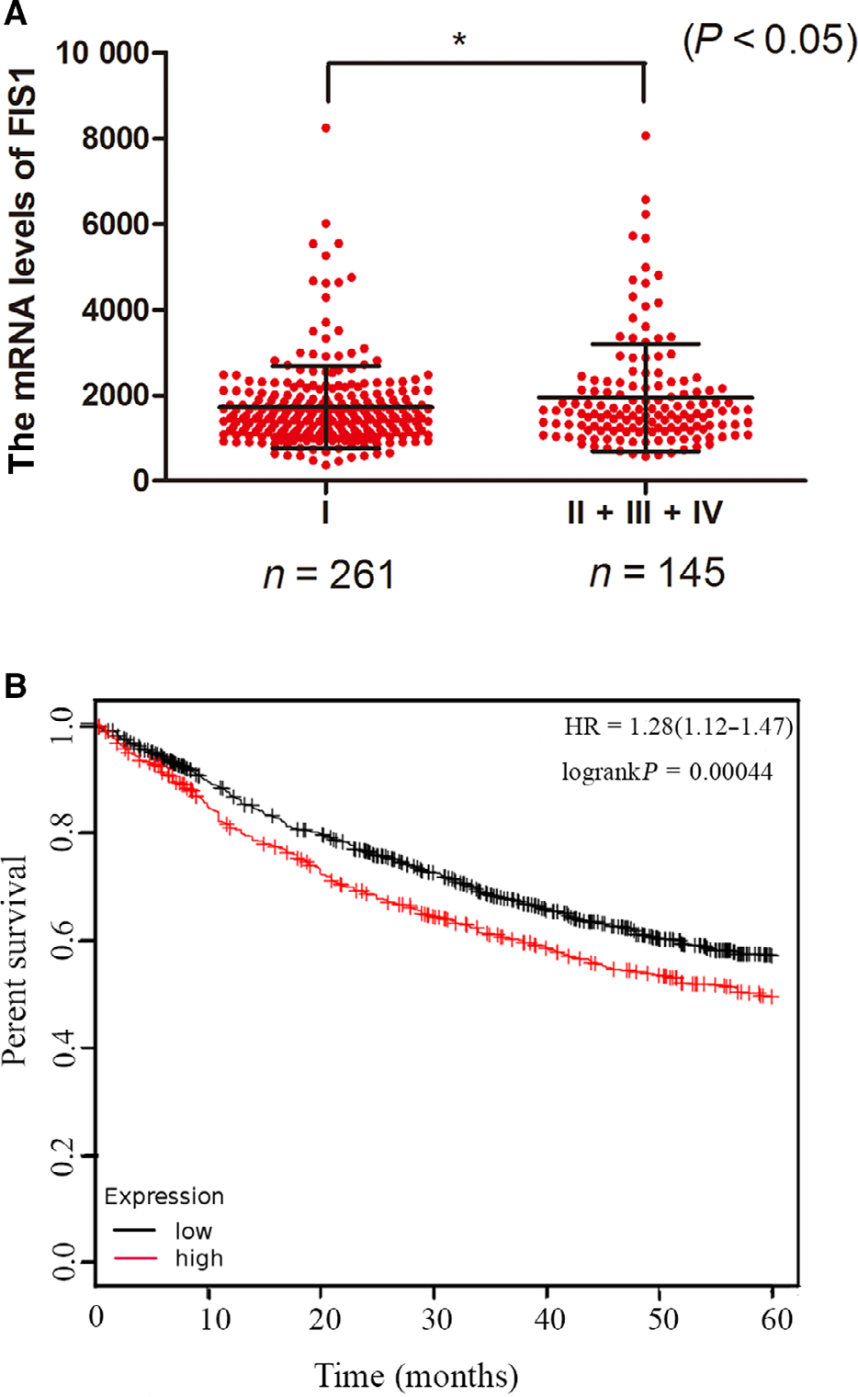
Clinical relevance analysis. (A) mRNA expression of FIS1 in different stages of lung adenocarcinoma. (B) Kaplan–Meier curve of the FIS1 low group and FIS1 high group. *P < 0.05.

To determine the clinical relevance of our findings in lung adenocarcinoma, we analyzed clinical data from lung adenocarcinoma patients in the The Cancer Genome Atlas (TCGA) database. We found that the expression of FIS1 was lower in stage I lung adenocarcinoma compared to that of stage II/III/IV lung adenocarcinoma (Fig. 5A). Kaplan–Meier plotter analysis revealed that patients with low FIS1 expression had significantly better survival (Fig. 5B). These results indicated that FIS1 may be combined with other factors to provide a more accurate prognosis for lung cancer progression. The significance of this clinical finding requires further investigation and validation.

## Discussion

In the present study, using the A549‐SD non‐small cell lung carcinoma stem cell model that we have developed and characterized [36], we have shown that activation of mitophagy by the *Fis1* gene augments the stemness of lung CSC. Specifically, we have made several observations that have not been reported previously.

First, differences exist between the mitochondrial traits of lung CSCs and non‐stem lung cancer cells. Lung CSCs have a higher mitochondrial content and a smaller mitochondrial size, as well as a denser peri‐nuclear distribution, compared to non‐stem counterparts. In addition, lung CSCs have lower ROS and higher mitochondrial membrane potential, consistent with prior reports in liver CSCs, leukemia CSCs and lung CSCs [37, 38]. However, previous reports did not use stable and purified CSCs; thus, a mechanistic understanding of such differences with respect to the regulation of the stemness of CSCs has not been achieved.

Second, we investigated the mechanism underlying the differences in the mitochondria features between lung A549‐SD CSCs and parental A549 cells. We found that mitochondrial fission gene *Fis1* is expressed at a higher level in A549‐SD CSCs compared to parental A549 cells. We then made the hypothesis that a mitochondrial fission gene such as *Fis1* regulates the stemness of lung CSCs. Pei *et al*. [31] showed that in leukemia CSCs, the highly expressed FIS1 could regulate the stemness of leukemia CSCs by mitophagy. In the present study, using the stable A549‐SD CSC model, we showed that, upon *Fis1* silencing, mitochondrial division was inhibited and the stemness of A549‐SD was also attenuated. This set of observations indicates that FIS1 augments the stemness of CSCs by promoting mitochondrial division. To our knowledge, our present study, for the first time, has demonstrated the direct regulation of the stemness of CSCs by FIS1. However, the mechanisms regulating FIS1 expression remain need to be elucidated. Furthermore, the specific mechanism underlying the regulation of mitophagy by FIS1 has not been investigated.

Third, we determined the clinical relevance of FIS1 in lung adenocarcinoma by analyzing the relationship between the expression of FIS1 in lung cancer patients and the clinical stage of lung cancer in the TCGA database. We found that there is an inverse relationship between FIS1 expression and the clinical stage of lung cancer. Furthermore, high FIS1 expression confers a shorter overall survival. Clinical validation studies are required to confirm the significance of FIS1 expression.

## Materials and methods

### Animals

BALB/c nude mice were purchased from the Experimental Animal Centre of Chongqing Medical University (Chongqing, China). Animal studies were conducted in accordance with an approved protocol and with the institutional animal welfare guidelines of Chongqing Medical University.

### Reagents for inhibiting and inducing mitophagy

The mitophagy inhibitor Mdivi‐1 (HY‐15886) and the inducer CCCP (HY‐100941) were purchased from MedChemExpress (Monmouth Junction, NJ, USA).

### Cell culture

A549 cells was purchased from the cell bank of the Shanghai Institute of Life Sciences, Chinese Academy of Sciences (Shanghai, China). A549 cells were cultured with RPMI 1640 culture medium that contained 1% amphotericin B, 1% penicillin‐streptomycin and 10% fetal bovines serum. The derivative CSC A549‐oncosphere cells (A549‐SD) were isolated and characterized as described previously [36] and the stemness of A549‐SD cells had also been verified [32]. Cells were cultured in Dulbecco’s modified Eagle's medium/F12 1 : 1 culture medium with 2% B27, 1% amphotericin B and 1% penicillin‐streptomycin. All cells were kept in the incubator with 5% CO_2_ and constant humidity at 37 °C.

### Six‐well plate serial spheroid formation assay

In this assay, single‐cell suspensions were plated at 1000 cells/well in a six‐well plate with 2 mL of culture medium. After 1 week in culture, clonogenic spheroids containing more than 50 cells were counted under microscopy. Spheroid cultures were then collected and single‐cell suspensions were prepared for setting up the second round of the assay. The assay was repeated for three consecutive rounds.

### Ninety‐six‐well plate single‐cell cloning assay

Single cell suspension was prepared and one cell was added into a well with 100 μL of culture medium. Single‐cell seeding in each well was confirmed by examination under a microscope and wells containing a single cell were marked. After culturing at 37 °C in 5% CO_2_ for 1 week, colonies exceeding 50 cells were counted.

### Reverse transcriptase‐PCR (RT‐PCR)

Total RNAs were isolated with Trizol agent (Thermo Fisher). In brief, 1 mL of Trizol agent were added into the Eppendorf tube containing cells, then incubated on the ice for 10 min, followed by the addition of 200 μL of chloroform. The mixture was centrifuged at 4 °C for 15 min followed by the addition of isoamyl alcohol and then incubation on the ice for 10 min. Finally, the mixture was centrifuged at 4 °C for 10 min followed by the addition of 70% ice‐cold ethanol and then another centrifugation in at 4 °C for 10 min. The pellet was dissolved using 20–30 μL of diethyl pyrocarbonate water. RT‐PCR was conducted using Prime Script RT Master Mix (Takara, Kyoto, Japan) in accordance with the manufacturer's instructions.

### Packaging of shRNA lentivirus

The small interfering RNA (siRNA) of FIS1 was purchased from GenePharma (Suzhou, China). Plasmid construction and lentivirus packaging were also prepared and supplied by GenePharma. Lentivirus‐mediated short hairpin RNA was obtained using H1/GFP&Puro (GenePharma).

### Real‐time quantitative PCR

The reaction volume for the quantitative PCR was 10 µL, which comprised 5 µL of Master Mix agent (Takara), 0.8 µL of cDNA, 0.8 µL of primer and 3.4 µL of diethyl pyrocarbonate water. Triplicates were set up for every sample. The PCR was conducted in accordance with the manufacturer's instructions. The sequences of PCR primers are provided in Table 1.

**Table 1:** PCR primer sequences

Gene name	Forward primers	Reverse primers
*Tbp*	CCGTGAATCTTGGCTGTAAAC	CAGTTGTCCGTGGCTCTCTT
*Bmi1*	ATCCCCACTTAATGTGTGTCCT	CTTGCTGGTCTCCAAGTA ACG
*Drp1*	ATCCAGCTGCCTCAAATCGT	TCTGCTTCCACCCCATTTTCT
*Mfn1*	GAGCGGAGACTCATAATGGCA	GTGGCTATTCGATCAAGTTCCG
*Mfn2*	AGCGGAGACTCATAATGGCA	TGGCTATTCGATCAAGTTCCG
*Opa1*	CTGTGGCCTGGATAGCAGAA	TGCTTCGTGAAACCAGATGT
*Mff*	CCGGGACCCTCCTGTGG	GACACTGCTTTTCCAGAAATGTG
*FIS1*	ATCCGTAAAGGCATCGTGCT	TCGTATTCCTTGAGCCGGTAG

### Western blotting

The present study used 12% resolving gel and 6% condensing gel. The voltage of electrophoresis was 120 V, whereas the current for member transfer was 350 mA. After transfer, the membrane was blocked with 5% BSA (BD, Franklin Lakes, NJ, USA) and then incubated with the primary antibody at 4 °C for 12 h, followed by incubation with the secondary antibody at room temperature for 1 h. The results were acquired using electrophoresis gel imaging (Bio‐Rad, Hercules, CA, USA).

### Subcutaneous tumor transplantation in nude mice

1 × 10^5^ cells were injected to each side of the hinder leg of each nude mouse. After 2 weeks, mice were killed and tumors were obtained and weighed.

### Immunofluorescence

Cells were collected, fixed with ice‐cold 70% ethanol for 30 min on the slide and treated with hydrochloric acid for 1 h. After washing with phoshate‐buffered saline three times, the slides were blocked with 10% BSA (BD) solution at 37 °C for 1 h. Thereafter, cells were incubated with the primary antibody at 4 °C for 12 h, followed by incubation with the secondary antibody at 37 °C for 1 h. Images were taken using a confocal microscope (Leica, Wetzlar, Germany).

### Clinical data analysis

mRNA expression of FIS1 in lung adenocarcinoma was evaluated by TCGA Research Network (http://cancergenome.nih.gov). To analyze the survival of patients with lung adenocarcinoma, patient samples were analyzed using Kaplan–Meier plotter analysis (http://kmplot.com).

### Statistical analysis

Data were analyzed by one‐way analysis of variance using prism, version 8 (GraphPad Software Inc., San Diego, CA, USA) and are presented as the mean ± SD. *P* < 0.05 was considered statistically significant. The size of the mitochondria was measured using image j.

## Conflict of interest

The authors declare no conflict of interest.

## Data accessibility

The analysed data sets generated during the present study are available from the corresponding author on reasonable reasonable request.

## Author contributions

DL and ZS conceived and designed the project. TY, JL and BZ acquired the data. DL and QZ analysed and interpreted the data. DL and ZS wrote the paper. JW and HRX improved the language of the manuscript and also reviewed the manuscript.
